# Combined DNase and Proteinase Treatment Interferes with Composition and Structural Integrity of Multispecies Oral Biofilms

**DOI:** 10.3390/jcm9040983

**Published:** 2020-04-01

**Authors:** Lamprini Karygianni, Thomas Attin, Thomas Thurnheer

**Affiliations:** Clinic of Conservative and Preventive Dentistry, Center of Dental Medicine, University of Zurich, 8032 Zurich, Switzerland; Lamprini.Karygianni@zzm.uzh.ch (L.K.); Thomas.Attin@zzm.uzh.ch (T.A.)

**Keywords:** supragingival in vitro biofilm, extracellular polymeric substances (EPS), DNase I, proteinase K, CLSM, FISH

## Abstract

Modification of oral biofilms adhering to dental hard tissues could lead to new treatment approaches in cariology and periodontology. In this study the impact of DNase I and/or proteinase K on the formation of a simulated supragingival biofilm was investigated in vitro. Six-species biofilms were grown anaerobically in the presence of DNase I and proteinase K. After 64 h biofilms were either harvested and quantified by culture analysis or proceeded to staining followed by confocal laser scanning microscopy. Microbial cells were stained using DNA-dyes or fluorescent in situ hybridization. Exopolysaccharides, eDNA and exoproteins were stained with Calcofluor, anti-DNA-antibody, and Sypro^TM^ Ruby, respectively. Overall, results showed that neither DNase I nor proteinase K had an impact on total colony-forming units (CFUs) compared to the control without enzymes. However, DNase I significantly suppressed the growth of *Actinomyces oris*, *Fusobacterium nucleatum*, *Streptococcus mutans*, *Streptococcus oralis* and *Candida albicans*. Proteinase K treatment induced significant increase in *S. mutans* and *S. oralis* CFUs (*p* < 0.001), whereas *C. albicans* and *V. dispar* showed lower CFUs compared to the control. Interestingly, confocal images visualized the biofilm degradation caused by DNase I and proteinase K. Thus, enzymatic treatment should be combined with conventional antimicrobial agents aiming at both bactericidal effectiveness and biofilm dispersal.

## 1. Introduction

Oral infectious diseases display the consequences of dynamic interactions between microorganisms, their host and the host’s diet, leading to microbial colonization of oral surfaces and the establishment of pathogenic biofilms [1]. Biofilms formed on either tooth or dental material surfaces are known as oral biofilms and have been clearly recognized as a virulence factor in several oral infectious diseases, including dental caries, periodontitis and endodontic infections [2]. Biofilms are defined as “aggregates of micro-organisms in which the associated cells are frequently embedded in a self-produced matrix of extracellular polymeric substances (EPS) that are adherent to each other and/or a surface [3]. The EPS consists of exopolysaccharides, nucleic acids (eDNA, eRNA), proteins and lipids [3]. The composition and structure of EPS varies according to the host, presence of nutrients/substrates, type of microorganisms, and local mechanical factors e.g., shear stress [4]. The EPS promotes microbial adhesion on biotic and abiotic surfaces.

The most prominent EPS functions include adhesion, cohesion, scaffolding, mechanical stability, protection and dispersal [5]. These EPS can promote and strengthen cell adhesion to solid substrates, coaggregation and cohesion among different microbial cells, eventually leading to development of microcolonies [6]. Upon microbial attachment, EPS is produced and keeps microbial cells in close proximity enabling intercellular interactions within the biofilm mass [3]. The EPS matrix also constitutes a mechanically stable and complex chemical microenvironment that is substantial for the biofilm lifestyle [7]. Furthermore, EPS also increases biofilm resistance to diverse antimicrobials and host defenses [8,9]. In the meanwhile, it is clear that biofilms contain multi EPS-framed heterogeneous milieus able to alter local gene expression, metabolic activity and, importantly, intercellular signaling between distinct cell clusters or different species within the biofilm mass [10,11]. Finally, the EPS can act as a diffusion-limiting barrier against various antimicrobials resulting in limited drug access into the deeper layers of the biofilm, and thus, low antimicrobial effectiveness of cationic antimicrobials e.g., chlorhexidine (CHX) [12]. The fact that positively charged antimicrobial agents bind to negatively charged EPS components can lead to restricted penetration of molecules through biofilms, and thus enhance antimicrobial biofilm resistance [9], thereby allowing for the inactivation or degradation of disinfectants by matrix enzymes [13].

Over the years, several studies in the field of endodontology have provided an excellent source of information about the disinfection of root canals by solely eliminating pathogens [14,15,16,17]. However, the fundamental role of biofilm matrix to combat biofilm-related diseases has been overlooked so far. The need for the development of EPS-targeted antibiofilm therapies is crucial, since in several cases, biofilm infections do not respond to conventional antimicrobial treatment [9,18]. The integral role of eDNA in the establishment of mechanical stability within biofilms has been pointed out in recent reports [19,20,21]. The eDNA is either actively secreted or produced by controlled cell lysis, which is associated with microbial competition [22,23]. The ability of eDNA to promote microbial adhesion and coaggregation between microbes, including interspecies recognition [24,25], and to convey antimicrobial resistance [23] has been extensively explored so far. The importance of matrix proteins has been also underlined in several studies to date [26,27,28]. Enzymatic biofilm treatment with proteinase K has been applied to induce protein degradation in the matrix, thereby enhancing the discharge of nucleic acids [29]. By targeting eDNA, the enzyme DNase I has been also successfully utilized to assist the elimination of cystic fibrosis-related biofilms by antibiotics [30].

Modification of oral biofilms adhering to dental hard tissues could lead to new treatment approaches in cariology and periodontology. In this study, therefore, we focus on novel antibiofilm strategies including those targeting the vital structural and functional traits in EPS with emphasis on eDNA and proteins. The aim of this study was to assess the antibiofilm activity of two different concentrations of DNase I (0.001 mg/mL, 0.002 mg/mL), proteinase K (0.05 mg/mL, 0.1 mg/mL) and combinations thereof against an in vitro simulated six-species supragingival biofilm upon constant enzymatic treatment over a period of 64 h. In this study, confocal laser scanning microscopy (CLSM) was used to visualize the effects of enzymatic treatment with DNase I and proteinase K on the structural integrity and the composition of the treated biofilms. To our knowledge, structural and compositional alterations in multispecies biofilms following enzymatic treatment with DNase I, proteinase K or their combination has not been studied to date. The null hypothesis of this study is that neither DNase I nor proteinase K has an antibiofilm effect on multispecies biofilms in vitro.

## 2. Materials and Methods

### 2.1. In Vitro Biofilm Experiments

The procedures to produce six-species biofilms have been described in detail [31,32]. In brief, *Actinomyces oris* OMZ 745, *Candida albicans* OMZ 110, *Fusobacterium nucleatum* KP-F2 (OMZ 596), *Streptococcus oralis* SK 248 (OMZ 607), *Streptococcus mutans* UA159 (OMZ 918), and *Veillonella dispar* ATCC 17748^T^ (OMZ 493) were used for biofilm formation. All strains were maintained on Columbia blood agar. Prior to the onset of biofilm experiments, all strains were transferred into modified fluid universal medium (mFUM) [33] and incubated under the same conditions anaerobically at 37 °C for two cycles of precultures for16 h and 8 h, respectively. For the production of the inoculum all strains were adjusted to a defined optical density (OD_550_ = 1.0) and mixed in equal volumes.

Biofilms were cultivated in 24-well polystyrene cell culture plates on sintered hydroxyapatite (HA; Ø 9mm, Clarkson Chromatography Products, Inc., South Williams-port, PA 17702, USA) that had been preconditioned for pellicle formation in whole unstimulated pooled saliva (in the following termed saliva) for 4 h. The processing of batches of saliva has been described in detail by Guggenheim et al. [33]. The growth medium contained 70% saliva, 30% mFUM supplemented with Sørensen’s buffer (final pH 7.2) as well as DNase I (0.001 mg/mL or 0.002 mg/mL) or proteinase K (0.05 mg/mL or 0.10 mg/mL) or a combination of DNase I + proteinase K (0.001 mg/mL + 0.05 mg/mL or 0.002 mg/mL + 0.10 mg/mL, respectively). The concentrations of DNase I and proteinase K have been determined in preliminary experiments (unpublished data). Control experiments contained neither DNase I nor proteinase K. The carbohydrate concentration of mFUM was either 0.3% glucose (0 to 16 h of biofilm cultivation) or 0.15% glucose and 0.15% sucrose (16 h to 64 h). To initiate a biofilm experiment, disks were covered with 1.6 mL of growth medium and 200 µL of inoculum. The medium was changed after 16 h and 40 h. In order to remove non-adherent microorganisms, biofilms were dipped three times in saline after 16 h, 20 h and 24 h as well as after 40 h, 44 h and 48 h. After 64 h of biofilm growth, biofilms were either harvested for culture analyses by vigorous vortexing in 1 mL of 0.9% NaCl or proceeded to staining and CLSM (see below).

For culture analyses, the hydroxyapatite disks were vortexed vigorously for 1 min in 1 mL of 0.9% NaCl to harvest the adherent biofilms. After vortexing the harvested biofilms were sonicated at 30 W for 5 s (Sonifier B-12, Branson Ultrasonic, Urdorf, Switzerland) to ensure that the bacteria were dispersed. The resulting bacterial suspensions were serially diluted in 0.9% NaCl Of each serial dilution, 50-μL aliquots were plated on Columbia blood agar (CBA) base (Oxoid Ltd., Basingstoke, UK) supplemented with 5% whole human blood to estimate total colony-forming units (CFUs). To determine the species-specific bacterial numbers, selective agars were used to determine the CFUs for the species of the biofilms as described earlier [33,34]. In brief, CBA plates were used to obtain total bacterial counts and to enumerate *A. oris* and *V. dispar*; differential counting of *S. mutans* and *S. oralis* was accomplished with the use of Mitis Salivarius Agar (Difco Laboratories, Inc., Detroit, MI) supplemented with 0.001% (*w*/*v*) sodium tellurite, whereas selective growth of *F. nucleatum* was achieved with Fastidious Anaerobe Agar (Chemie Brunschwig, Basel, Switzerland) and BIGGY Agar (BBL, Becton Dickinson, Allschwil, Switzerland) was used to enumerate *C. albicans*. Agar plates were incubated at 37 °C for 72 h. Species identification was achieved by observation of colony morphology.

### 2.2. Staining of Biofilms

Prior to staining, biofilms were fixed in 1 mL of 4% paraformaldehyde + RNase inhibitor (RNAi) for two hours at 4–8 °C. After fixation, discs were washed in 500 μL 0.9% NaCl + RNase Inhibitor and dabbed off on a paper towel. In order to permeabilize Gram-positive bacteria biofilms were pretreated with 1 mg/mL lysozyme solution (Sigma, Buchs, Switzerland; 70V000 U/mL) in 0.1 M Tris-HCl, pH 7.5, 5 mM EDTA for 8 min at room temperature and rinsed with 0.9% saline.

Total DNA was stained with a mixture of 3 μM YoPro 1 iodide (Invitrogen, Basel, Switzerland) and 15 μM Sytox green (Invitrogen) in nanopure water for 30 min or with 0.5 µg/mL 4′,6-diamidino-2-phenylindole (DAPI) (SERVA Electrophoresis GmbH, Heidelberg, Germany) in nanopure water for 5 min at room temperature in the dark. *Streptococcus mutans* and *S. oralis* cells were specifically stained using fluorescence in situ hybridization (FISH) following earlier described protocols [35,36]. In brief, prehybridization (15 min, 46 °C) was performed in 500 μL hybridization buffer with 25% formamide in the absence of any oligonucleotide probes. Thereafter, 500 μL of hybridization buffer (25% formamide) was used for each biofilm, supplemented with the species-specific fluorescein isothiocyanate (FITC)-labeled probe MIT447 (5′-CACYCGTTCTTCTCTTACA-3′) and Cy3-labeled probe MUT590 (5′-ACTCCAGACTTTCCTGAC-3′) to stain *S. oralis* and *S. mutans*, respectively, at a concentration of 20 ng/μL. The incubation time for the hybridization was at least 3 h at 46 °C in the dark. After the incubation, biofilms were transferred into washing buffer preheated to 48 °C and incubated for 20 min at this temperature.

The biofilm matrix was stained after FISH. Extracellular polysaccharides were stained by incubating biofilms with Calcofluor (Sigma; 10 µg/mL solution in 10 mM sodium phosphate, pH 7.5) for 30 min at room temperature in the dark. Extracellular DNA (eDNA) was stained with Cy3-streptavidin labelled anti-DNA-antibody (Sigma-Aldrich, Buchs, Switzerland) according to the manufacturer’s recommendations, and extracellular proteins were stained with Sypro^TM^ Ruby according to the manufacturer’s protocols.

After staining, the samples were embedded upside down on chamber slides in 100 μL of Mowiol [37].

### 2.3. Confocal Laser Scanning Microscopy (CLSM)

CLSM was conducted using a Leica TCS SP5 microscope (Leica Microsystems, Wetzlar, Germany) provided by the Centre for Microscopy and Image Analysis of the University of Zurich. For the imaging of the biofilms on hydroxyapatite, the slightly modified procedure as described before [38], was performed. Briefly, the used lasers were an ultraviolet (UV) laser at 405 nm excitation, an Argon laser at 488 nm excitation, a diode pumped solid state (DPSS) diode laser at 561 nm, and a helium-neon laser at 633 nm excitation. Furthermore, filters were adjusted at 430–470 nm to detect DAPI, at 500–540 nm for Yo Pro 1/Sytox green and FITC, at 570–600 nm for Cy3, and at 660–710 nm for Sypro^TM^ Ruby. Biofilms were scanned sequentially in steps of 1 μm thickness. Finally, the images were processed using Imaris 8.3 (Bitplane, Zurich, Switzerland).

### 2.4. Statistical Analysis

Three individual experiments were performed and each group represented in triplicate biofilm cultures per experiment. As a result, statistical analysis was performed on nine individual data points, coming from the nine individual biofilm cultures per experimental group. Two-way analysis of variance (ANOVA) was used to analyze the difference in bacterial cells per biofilm between the control group (standard nine-species biofilm) and the six additions of endodontic strains. Tukey’s multiple comparisons test was used for correction. Missing values were ascribed the lowest detection limit value of the assay to allow for logarithmic transformation. Statistics have been implemented using GraphPad Prism (version 7) with the intent of comparing the species’ total cell counts within the different biofilm formations (significance level *p* < 0.05).

## 3. Results

### 3.1. DNase I Significantly Suppressed the Growth of A. oris, F. nucleatum, S. mutans, S. oralis and C. albicans

**Figure 1:**
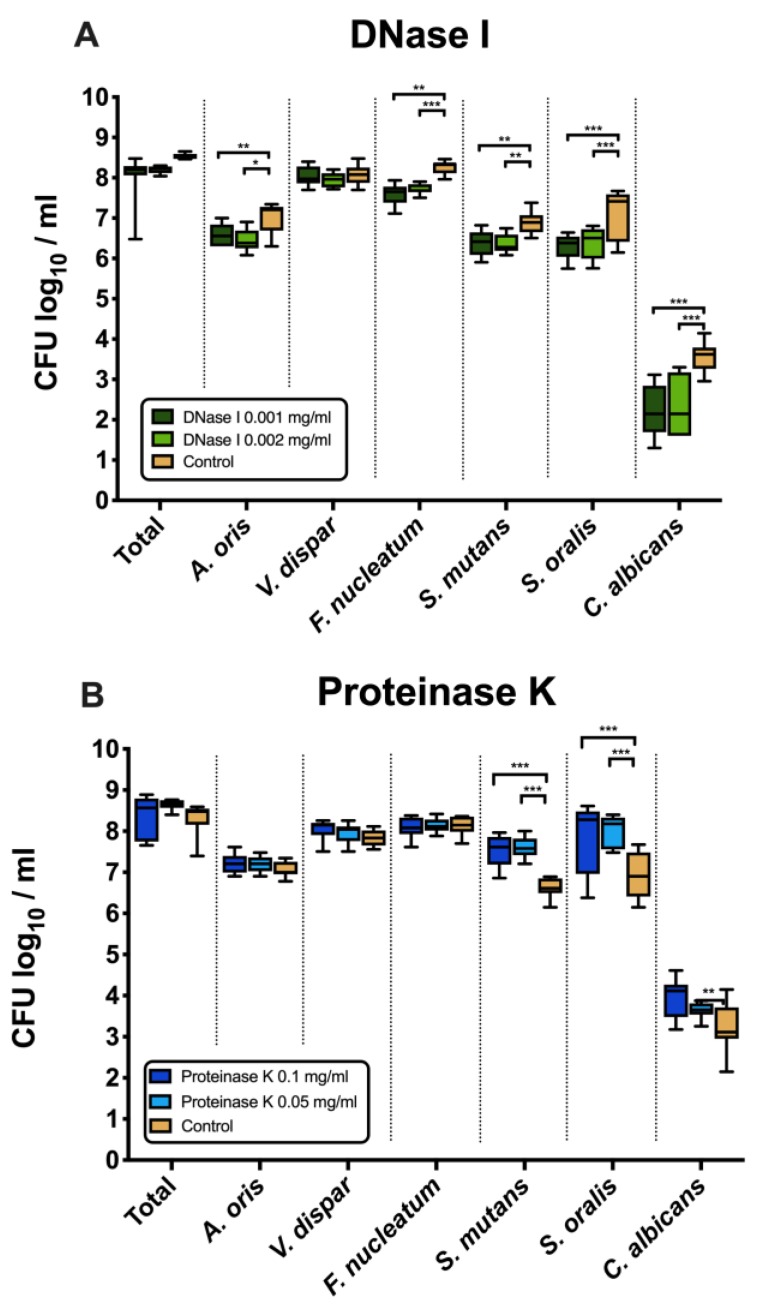
Box plots depicting the colony-forming units (CFUs) of six-species oral biofilms after exposure to DNase I (0.001 mg/mL, 0.002 mg/mL) (**A**) or proteinase K (0.05 mg/mL, 0.1 mg/mL) (**B**), respectively. Untreated biofilms were also tested as negative controls. The CFUs are shown on a log_10_ scale per millilitre (Log_10_/_mL_). Statistically significant differences are indicated with asterisks (* *p* < 0.030, ** *p* < 0.006, *** *p* < 0.0001).

Figure 1A shows the effects of DNase I on the log_10_ counts of six-species supragingival biofilms grown over 64 h in vitro. No significant differences could be found in regard with the effects of the low (0.001 mg/mL) versus high (0.002 mg/mL) tested DNase I concentration. Constant incubation of the biofilms with two different concentrations of DNase I (0.001 mg/mL, 0.002 mg/mL) had also no effect on the total bacterial counts. However, the log_10_ counts of five individual microbial species decreased significantly in a dose-independent manner after DNase I treatment. In particular, *A. oris* exhibited means of 6.45 ± 0.27 CFU (0.001 mg/mL DNase I, *p* = 0.002) and 6.58 ± 0.26 CFU (0.002 mg/mL DNase I, *p* = 0.026) in the log_10_ scale, which is a slightly, yet significantly reduced microbial growth compared with the untreated negative control (mean, 6.99 ± 0.36). The counts of *F. nucleatum* (0.001 mg/mL DNase I, 7.71 ± 0.12 log_10_ CFU, *p* = 0.002; 0.002 mg/mL DNase I, 7.58 ± 0.25 log_10_ CFU, *p* = 0.0001) and *C. albicans* (0.001 mg/mL DNase I, 2.35 ± 0.72 log_10_ CFU, *p* = 0.0001; 0.002 mg/mL DNase I, 2.19 ± 0.62 log_10_ CFU, *p* = 0.0001) also decreased after DNase I treatment compared to the negative control. Finally, treatment with DNase I had a negative impact on the streptococcal growth of *S. mutans* (0.001 mg/mL DNase I, 6.37 ± 0.23 log_10_ CFU, *p* = 0.005; 0.002 mg/mL DNase I, 6.36 ± 0.31 log_10_ CFU, *p* = 0.004) and *S. oralis* (0.001 mg/mL DNase I, 6.38 ± 0.37 log_10_ CFU, *p* = 0.0001; 0.002 mg/mL DNase I, 6.30 ± 0.29 log_10_ CFU, *p* = 0.0001) as compared to the streptococci in the untreated biofilms.

### 3.2. Counts of S. mutans, S. oralis and C. albicans Increased after Treatment with Proteinase K

Figure 1B displays the microbial growth rates of in vitro six-species supragingival biofilms incubated for 64 h with two different concentrations of proteinase K (0.05 mg/mL, 0.1 mg/mL).

Independent of the tested proteinase K concentration, all proteinase K-treated biofilms showed microbial growth comparable to that of the negative control. Nevertheless, the log_10_ counts of three individual microbial species increased substantially after biofilm treatment with proteinase K. Specifically, both treatments with 0.05 mg/mL (mean, 7.59 ± 0.25 log_10_ CFU; *p* = 0.0001) and 0.1 mg/mL proteinase K (mean, 7.52 ± 0.40 log_10_ CFU; *p* = 0.0001) yielded a low, yet significant log_10_ CFU increase in *S. mutans* counts compared with the untreated negative control (mean, 6.62 ± 0.23 log_10_ CFU). Similarly, a mild elevation of *S. oralis* log_10_ CFU counts was induced after biofilm incubation in 0.05 mg/mL (mean, 8.02 ± 0.38 log_10_ CFU; *p* = 0.0001) and 0.1 mg/mL proteinase K (mean, 7.84 ± 0.88 log_10_ CFU; *p* = 0.0001) as compared to the untreated biofilms (mean, 6.94 ± 0.55 log_10_ CFU). Finally, the low proteinase K concentration of 0.1 mg/mL (mean, 3.64 ± 0.19 log_10_ CFU; *p* = 0.0003) yield a minor increase in the log_10_ CFU counts of *C. albicans* compared with the negative control (mean, 3.25 ± 0.59 log_10_ CFU).

### 3.3. Combined Treatment with DNase I and Proteinase K Induced a Significant Increase in Total Bacterial, S. mutans, and S. oralis Counts

**Figure 2:**
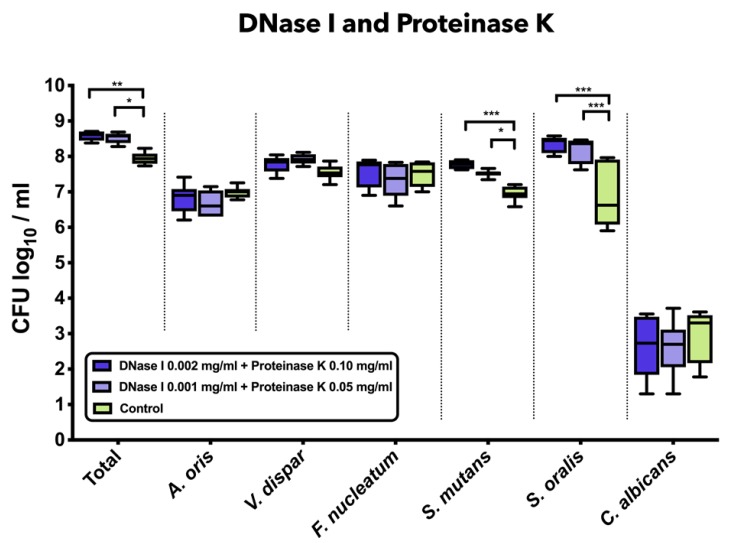
Box plots depicting the colony-forming units (CFUs) of six-species oral biofilms after simultaneous exposure to DNase I (0.001 mg/mL, 0.002 mg/mL) and proteinase K (0.05 mg/mL, 0.1 mg/mL). Untreated biofilms were also tested as negative controls. The CFUs are shown on a log_10_ scale per millilitre (Log_10_/_mL_). Statistically significant differences are indicated with asterisks (* *p* < 0.030, ** *p* < 0.006, *** *p* < 0.0001).

Figure 2 demonstrates the impact of the combined treatment with DNase I and proteinase K on the log_10_ counts of six-species supragingival biofilms grown over 64 h in vitro. The application of 0.001 mg/mL DNase I and 0.05 mg/mL proteinase K (mean, 8.52 ± 0.14 log_10_ CFU; *p* = 0.0124) or 0.002 mg/mL DNase I and 0.1 mg/mL proteinase K (mean, 8.58 ± 0.13 log_10_ CFU; *p* = 0.0003) had a low, yet significant effect on total microbial counts compared to the negative controls (mean, 7.95 ± 0.16 log_10_ CFU). Treatment with 0.001 mg/mL DNase I and 0.05 mg/mL proteinase K (mean, 7.52 ± 0.09 log_10_ CFU; *p* = 0.0134) or 0.002 mg/mL DNase I and 0.1 mg/mL proteinase K (mean, 7.76 ± 0.11 log_10_ CFU; *p* = 0.0002) had a positive impact on the streptococcal growth of *S. mutans* as compared to the untreated biofilms. Accordingly, a mild, yet significant increase of *S. oralis* log_10_ CFU counts was induced after biofilm incubation in 0.001 mg/mL DNase I and 0.05 mg/mL proteinase K (mean, 8.17 ± 0.34 log_10_ CFU; *p* = 0.0001) or 0.002 mg/mL DNase I and 0.1 mg/mL proteinase K (mean, 8.32 ± 0.22 log_10_ CFU; *p* = 0.0001) as compared to the untreated biofilms (mean, 6.84 ± 0.87 log_10_ CFU).

### 3.4. Combined Treatment with DNase I and Proteinase K Affected the Structural Integrity and Spatial Distribution of Biofilms

**Figure 3:**
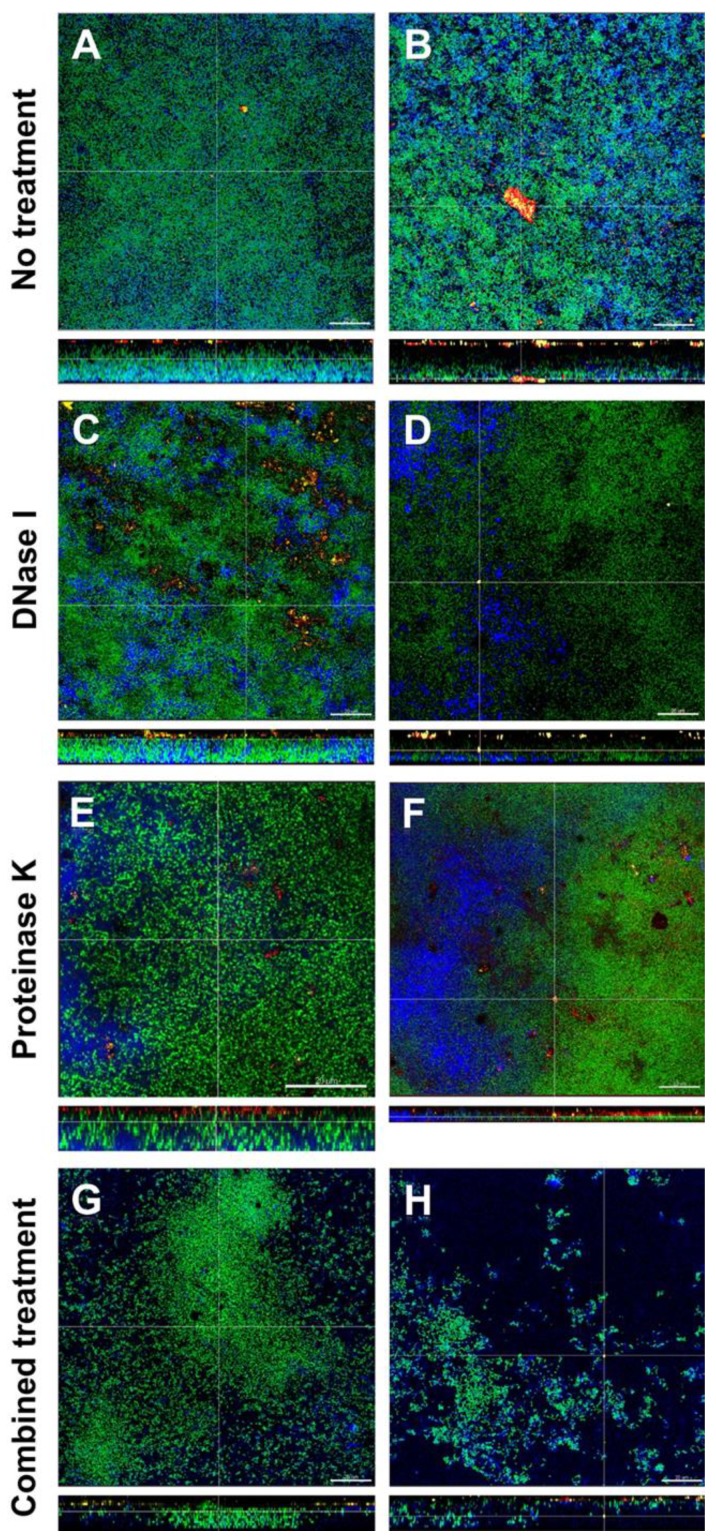
Representative confocal laser scanning microscopy (CLSM) images of six-species oral biofilms after no treatment (**A,B**), or exposure to 0.001 mg/mL DNase I (**C**), 0.002 mg/mL DNase I (**D**), 0.05 mg/mL proteinase K (**E**), 0.1 mg/mL proteinase K 0.1 mg/mL (**F**), or combined treatment with 0.001 mg/mL DNase I and 0.05 mg/mL proteinase K (**G**), or with 0.002 mg/mL DNase I and 0.1 mg/mL proteinase K (**H**), respectively. Bacteria appear green due to DNA-staining with Yo Pro 1/Sytox Green; extracellular polysaccharides are stained blue with calcofluor; extracellular DNA appear red after staining with anti-DNA antibodies and streptavidin (Cy3); extracellular proteins appear yellow due to Sypro^TM^ Ruby. The biofilm base in the cross sections is directed towards the top view. Scale = 20 µm.

The effects of DNase I and proteinase K treatment of the in vitro biofilms is shown in representative CLSM images in Figure 3. In the left column the effects with low concentrated DNase I and proteinase K (0.001 mg/mL and 0.05mg/mL, respectively) are shown, the right column shows the effects with high concentrated DNase I and proteinase K (0.002 mg/mL + 0.10 mg/mL, respectively). Biofilm organisms are stained green, extracellular polysaccharides appear blue, eDNA is stained red and extracellular proteins yellow. In the untreated biofilms (control; Figure 3A,B), eDNA and extracellular proteins could be observed throughout the whole biofilm. Remarkably, eDNA and extracellular proteins seemed to form small or even large aggregates. In DNase I treated biofilms (Figure 3C,D), eDNA could only be observed in the biofilm treated with low concentrated DNase I (Figure 3C) but not in the biofilm treated with high concentrated DNase (Figure 3D), whereas extracellular proteins could be seen in both biofilms. Although no quantification was performed, both biofilms treated with proteinase K (Figure 3E,F) showed fewer extracellular proteins but contrary to DNase I treatment, where all eDNA seemed to be degraded, even high concentrated proteinase was not able to break down all extracellular proteins. However, proteinase K treatment resulted in less dense biofilms compared to the control. The combined action of DNase I and proteinase K (Figure 3G,H) again showed less eDNA and only a few remaining extracellular proteins (although no quantification was performed) but most notably the structural integrity of the biofilms was affected, especially in the biofilm treated with high concentrated DNase I and proteinase K which can be observed in Figure 3H. Compared to the control biofilm, the density and spatial distribution of the cells and extracellular matrix was substantially reduced and although no extracellular polysaccharide-degrading enzymes had been applied, the extracellular polysaccharides strongly decreased, too (Figure 3G,H).

### 3.5. Fluorescence In Situ Hybridization (FISH)/CLSM Images Highlighted the Dominance of Streptococci after Combined Treatment with DNase I and Proteinase K

**Figure 4:**
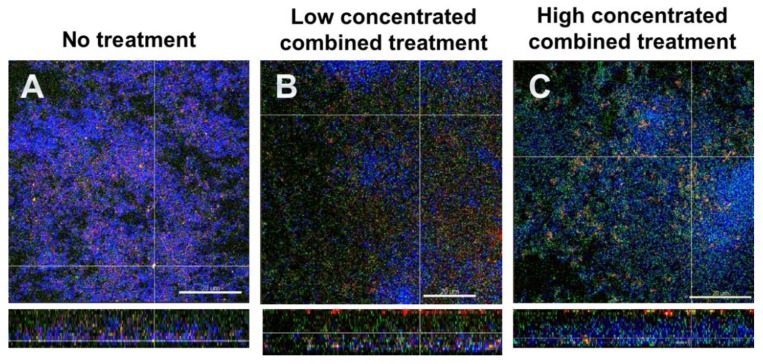
Representative CLSM images of six-species oral biofilms after no treatment (**A**), or combined treatment with 0.001 mg/mL DNase I and 0.05 mg/mL proteinase K (**B**), or with 0.002 mg/mL DNase I and 0.1 mg/mL proteinase K (**C**), respectively. After fluorescence in situ hybridization (FISH), microbial DNA appears blue due to DNA-staining with 4′,6-diamidino-2-phenylindole (DAPI); *S. oralis* is stained green with MIT447- fluorescein isothiocyanate (FITC); *S. mutans* appears red after staining with MUT590-Cy3; extracellular proteins appear yellow due to Sypro^TM^ Ruby. The biofilm base in the cross sections is directed towards the top view. Scale = 20 µm.

Figure 4 shows FISH-stained biofilms. Total cells appear blue due to DAPI staining, *S. mutans* is stained red, *S. oralis* green and extracellular proteins are stained yellow. Figure 4A depicts the untreated biofilm. The combined action of the effects with low concentrated DNase I and proteinase K (0.001 mg/mL and 0.05mg/mL, respectively) are shown in Figure 4B, and the effects with high concentrated DNase I and proteinase K (0.002 mg/mL + 0.10 mg/mL, respectively) are shown in Figure 4C. It is evident in particular that the combined action of high concentrated DNase I and proteinase K affected the numbers of streptococci, as in Figure 4C an increase in *S. oralis* cells is evident. Again, the enzyme-treated biofilms appeared less dense and seemed to have lost structural integrity.

## 4. Discussion

The present study attempted for the first time to establish an effective method for enzymatic inhibition of in vitro multispecies oral biofilm growth using DNase I, proteinase K or their combinations. Although no decrease of the total bacterial counts was detected after constant incubation of the biofilms with two different concentrations of DNase I (0.001 mg/mL, 0.002 mg/mL) or proteinase K (0.05 mg/mL, 0.1 mg/mL), the application of combined enzymatic therapy with DNase I and proteinase K resulted in a slight decrease of the total microbial counts compared to the negative controls. In general, all enzymatic treatment protocols resulted in the shift of the microbial composition within the multispecies biofilms. In particular, treatment with DNase I resulted in the decrease of facultative and obligate anaerobic microorganisms, while the application of proteinase K mainly promoted streptococcal growth. Most importantly, biofilm visualization by CLSM revealed the degradation of the matrix, which led to the loss of structural integrity and alteration of spatial distribution of biofilms after combined treatment with DNase I and proteinase K. The combined enzymatic treatment not only induced biofilm degradation, it also favored the growth of streptococci probably by reducing nutritional competition. The innovative contribution of this study is that, to the best of our knowledge, the enzymatic inhibition of multispecies oral biofilm growth in vitro is here studied for the first time in combination with DNase I and proteinase K. The null hypothesis of this study was rejected.

To study the bacterial colonization of tooth surfaces, hydroxyapatite slabs served as a representative substratum model with comparable physicochemical properties with that of human enamel. Another advantage of hydroxyapatite slabs is that they can be easily obtainable in large quantities. Other artificial tooth surfaces were avoided due to the alterations in biofilm composition when compared with biofilms attached to natural substrata [39]. The bacterial growth after the enzymatic treatment with DNase I, proteinase K, or both was quantified by the determination of CFU on agar media, which not only constitutes a representative cultural approach for assessing the antibiofilm effectiveness of the enzymatic therapy protocols but is also the most widely used technique to estimate biofilm cell viability. Based on the universal dilution series approach used to quantify cells, this technique is available in every microbiological laboratory. However, this method presents serious drawbacks and limitations [40] as it is time-consuming and labor-intensive, sometimes requiring days to perform enough replicates to obtain reproducible results. Moreover, the fraction of detached live cells may not be representative of the initial biofilm population and a subpopulation of biofilm cells can be viable but non-culturable (VBNC) and would not be detected by the CFU approach. Furthermore, since the biofilm requires suspension, errors can occur due to bacterial clumping and, if antimicrobial treatment was used, carryover can occur. An important consideration for choosing the culture technique, especially when antimicrobial treatment is involved, is that only live cells, capable of forming a colony, are counted. The problems of clumping have been overcome by sonicating the suspensions to ensure that the microorganisms were dispersed, and extensive washing of the biofilms in saline helped to minimize the carryover. Alternative methods of biofilm quantification are real-time polymerase chain reaction (PCR) and flow cytometry. The latter has been used to quickly and accurately determine biofilms cell viability [41], and although it allows differentiating between total, dead and VBNC, flow cytometry is definitively more expensive than CFU counting and, moreover, it does not allow microorganisms to be discriminated at the species level. Real time PCR on the other hand has been tested in earlier studies [42]. Although live and dead cells are counted with this technique, a PCR-based method using propidium monoazide can be applied to exclude or even quantify dead cells in oral multispecies biofilms [43]. Therefore, we decided to use the culture method for quantification of the supragingival biofilm model as previously described by Guggenheim et al. [33]. As far as CLSM imaging is concerned, the fluorescence signals from the in vitro oral biofilms were not affected by the autofluorescence of hydroxyapatite, although an earlier study had reported contradictory outcomes [44].

To date, a plethora of antimicrobial strategies have been applied for biofilm dispersal in dental medicine, and especially in regard with root canal disinfection [14,15,16,17]. However, due to several limitations of antimicrobials e.g., low effectiveness against mature biofilms, chemical treatment protocols can be supplemented by enzymatic methods. Enzymes such as DNases and proteinases can allow for the degradation of EPS, and thereby provide access of antimicrobials to the deeper biofilm layers, which are typically colonized by the most resistant microbial cells [45,46]. Besides DNase II, and TREX1 (or DNase III), DNase I is the major serum endonuclease that degrades eDNA in the extracellular matrix [47], and been combined with antibiotics [48], or clinically applied to disperse *P. aeruginosa* biofilms in cystic fibrosis patients [49]. In our report, targeting the eDNA with DNase I in the multispecies biofilm matrix had no impact on the total bacterial counts, but altered the bacterial composition and, thus, the spatial distribution of the bacterial species within the biofilm (Figure 1A). This effect may be attributed eventually to the active role of eDNA in gene transfer, which is facilitated by the close proximity of the microbial cells within the biofilm [50]. The fact that DNase I affected the growth of *A. oris*, *F. nucleatum*, *S. mutans*, *S. oralis* and *C. albicans* negatively, could be related to the function of eDNA as a nutrient source for immobilized biofilm cells [51]. Since the formation of oral biofilms reflects the ecological balance between different microbes colonizing the same microenvironment, our study showed that DNase I treatment can disturb this balance and may, therefore, be relevant for some combined clinical applications against biofilms. Although quorum sensing in multispecies biofilms has not been thoroughly investigated yet, it is possible that a still unknown quorum-sensing mechanism triggers a compositional shift within the biofilms after DNase I treatment to anticipate for the mechanical instability caused by the absence of eDNA [52].

Since proteins belong together with polysaccharides to the main constituents of EPS, diverse proteases such as Spl protease, proteinase K, and aureolysin seem to have high potential for matrix degradation, and thus, biofilm removal [53]. In previous studies, treatment with proteinase K induced a significant detachment of proteinaceous—rich in Bap proteins—*Staphylococcus aureus* biofilms [54,55]. Similarly, 200 μg/mL proteinase K triggered complete dispersal of *Listeria monocytogenes* biofilms in 5 min [56]. However, in another report, concentrations of proteinase K up to 32 μg/mL had no impact on planktonic growth of *S. aureus* cells [57]. In our study, despite the fact that constant treatment with proteinase K during biofilm formation for 64 h had no effect on total bacterial counts, it favored streptococcal growth of *S. mutans* and *S. oralis* (Figure 1B). This can be attributed to the fact that streptococci may not only need amino acids for biosynthetic processes to neutralize the acidity resulting from carbohydrate metabolism, but also as a nutrient source [58,59]. Another explanation for the proteinase K-mediated increase in streptococcal growth is that proteinase K presumably deactivates quorum-sensing- or competence-stimulating peptides, which may be involved in either streptococcal proliferation or interspecies competition within biofilms, respectively.

While the use of culture method allowed for the quantification of the microbial counts and the assessment of the microbial shift after the enzymatic inhibition of the biofilm growth, CLSM was used to examine the three-dimensional (3D) structure of biofilms. The combination with FISH and other staining procedures allows biofilm microorganisms to be stained specifically as well as the components of the biofilm matrix. To demonstrate the effects of DNase I and proteinase K on the structure of in vitro biofilms, we used DNA dyes such as Yo Pro 1/Sytox green or DAPI to stain the bulk of biofilm cells. The biofilm matrix components such as extracellular polysaccharides, eDNA, and extracellular proteins were stained with Calcofluor, Cy3-streptavidin labelled anti-DNA-antibody, and Sypro^TM^ Ruby, respectively. The combined application of DNase I and proteinase K affected also the biofilm infrastructure, by inducing less compact biofilms compared to untreated biofilms. Whitchurch et al. [60] investigated the effect of DNase I on biofilm formation using a flow-chamber system. Their results using *Pseudomonas aeruginosa* biofilms showed that the presence of DNase I in the medium prevented biofilm formation and the authors suggested that DNase I treatment might be beneficial as an early prophylactic measure to prevent biofilm establishment. In our study biofilms were cultured in the presence of DNase I which resulted in an efficient removal of eDNA. However, this effect was not so prominent when DNase treatment was made after biofilm formation as seen in previous experiments. Similar results were reported by Yu et al. [61] when DNase was applied on *E. faecalis* biofilms in an in vitro root canal system. In the same study it was demonstrated that DNase treatment affected the microstructure of the biofilms. Also, compared to the control, the volume of exopolysaccharides in the DNase-treated biofilm was significantly lower in this endodontic biofilm model [61]. Our study showed similar results, however, the reduction of exopolysaccharides was more evident when biofilms were treated with DNase I and proteinase K (Figure 3H). *Streptococcus pneumoniae* clinical isolates showed evidence of eDNA as demonstrated by in situ staining of the matrix with the eDNA stain PicoGreen [62]. Treatment of these isolates with DNase I resulted in a dose-dependent reduction of the pneumococcal biofilm biomass as we observed also in our in vitro biofilms.

Niazi et al. [63] investigated the dynamics of biofilm killing and disruption using 1% trypsin and 1% proteinase K on an endodontic multispecies biofilm model. Confocal images of live-dead stained biofilms showed that trypsin and proteinase K were effective in killing bacteria, as the viable counts were significantly lower than in the negative control. In another study, the same authors examined the synergistic effect of a disinfection regimen consisting of trypsin and proteinase K in combination with chlorhexidine on an endodontic multispecies biofilm model. Chlorhexidine used in combination with trypsin and proteinase K enhanced the biofilm killing effect of the entire disinfection regime [64]. In our study, proteinase K was able to remove most of the extracellular proteins and the treatment resulted in less dense biofilms compared to the control biofilm. A synergistic effect could be observed, when proteinase K was combined with DNase I which affected also the structural integrity of the biofilms (Figure 3G,H). Ali Mohammed et al. [65] tested DNase I and proteinase K on *Fusobacterium nucleatum* and *Porphyromonas gingivalis* biofilms after 0 h and 48 h of growth. However, DNase I and proteinase K had little effect on the biofilm matrix in the conditions used. In a study by Lim et al. [66] *Escherichia coli* biofilms were treated with DNase I, proteinase K and sodium hypochlorite. When enzymes were added to the preformed biofilms on a stainless steel substrate, none of the DNase I, proteinase K, or NaClO treatment alone significantly reduced the number of viable cells. However, the combined treatment using proteinase K followed by NaClO, showed a notable reduction of viable cells [66]. The role of eDNA and exoproteins on biofilm formation were investigated by George and Halami [67] by treating mature *Lactobacillus plantarum* biofilms with DNase I and proteinase K. While untreated biofilms were observed to possess significantly dense population of both live and dead cells, a considerable decrease in the cell density was evident in DNase I-treated and proteinase K-treated biofilms. The authors concluded that the examined biofilms comprised eDNA and exoproteins and that these matrix components are vital constituents of biofilms [67]. In our study, the combined application of DNase I and proteinase K not only led to a loss of biofilm integrity, it also favored growth of streptococci (see Figure 4C) probably by reducing nutritional competition between the biofilm organisms. Enzymatic treatment of the biofilms seemed to help disrupting the bacteria from the biofilm matrix; it would be interesting to see, whether such DNase I and proteinase K treated biofilms favor synergistic effects with antimicrobial agents. This, however, remains to be elucidated in future studies.

It should be noted that neither DNase I nor proteinase K treatment had a negative impact on total bacterial counts. This implies that both enzymes interact only with eDNA and extracellular matrix proteins, without penetrating the intact membranes of the microbial cells, thereby interfering solely with the structural integrity of the biofilms. So, despite the fact that DNase I and proteinase K can degrade biofilms effectively, they should be combined with conventional antimicrobial agents aiming at both bactericidal effectiveness and biofilm dispersal.
